# PPACK and Bivalirudin nanoparticles enable simultaneous imaging and potent inhibition of acute clotting

**DOI:** 10.1186/1532-429X-14-S1-O41

**Published:** 2012-02-01

**Authors:** Jacob W Myerson, John S Allen, Todd A Williams, Li He, Douglas M Tollefsen, Gregory Lanza, Shelton D Caruthers, Samuel A Wickline

**Affiliations:** 1 Biomedical Engineering, Washington University in St. Louis, Saint Louis, MO, USA; 2 Medicine, Washington University, Saint Louis, MO, USA

## Summary

Perfluorocarbon nanoparticles functionalized for thombin inhibition with Bivalirudin or PPACK were tested as an inhibitor of thrombin in vitro and in acute thrombosis models. The particles significantly inhibited occlusive arterial thrombi. The particles also manifested binding providing magnetic resonance contrast highlighting thrombi.

## Background

Acute thrombosis is currently addressed with a cocktail of anticoagulants, antiplatelet agents, and thrombolytics. Optimization of antithrombotics for clinical use remains a significant research challenge. Thrombin inhibitors represent a promising class of anticoagulant. Imaging contrast agents specific to thrombi would represent a boon to clinical intervention, enabling monitoring of thrombosis. In recent work, a thrombin-inhibiting perfluorocarbon nanoparticle (PFC NP), functionalized by the direct thrombin inhibitor, PPACK (Phe(D)-Pro-Arg-Chloromethylketone), was presented as a prototype for novel class of targeted antithrombotic. Here, an NP functionalized with Bivalirudin (BVR), was compared to BVR and the PPACK NP. Both particles were examined as agents for inhibition and imaging of acute thrombosis.

## Methods

PPACK or BVR were covalently attached to PFC NPs. Optical assay verified that PPACK and BVR selectivity and activity against thrombin was not diminished on the NPs. In vivo activity was assessed for PPACK NPs, PPACK, BVR, BVR NPs, heparin, non-functionalized NPs, or saline in C57BL6 mice subjected to laser injury of the carotid artery. Time to thrombotic occlusion of the injured artery was assessed via Doppler flow measurement. Selected arteries were excised to assess NP retention via 19F MR at 12T. The laser injury arterial thrombosis model was applied in cholesterol-fed rabbits to demonstrate 3T MRI of thrombosis following administration of PPACK NPs. 19F MRI of the arterial segment at 12T enabled mapping of the thrombus.

## Results

**Figure 1:**
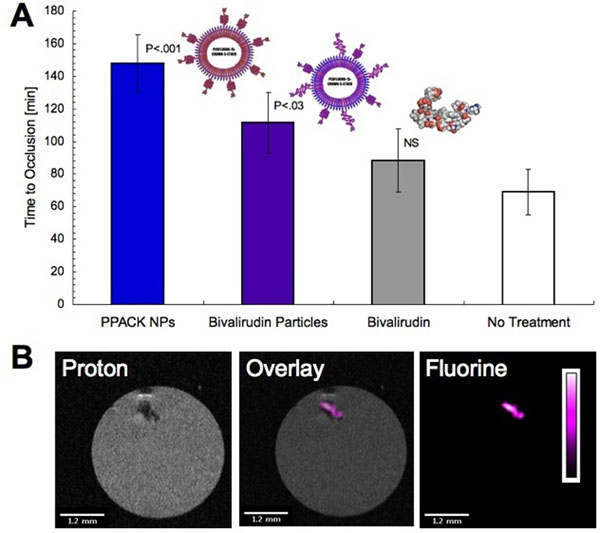


**Figure 2:**
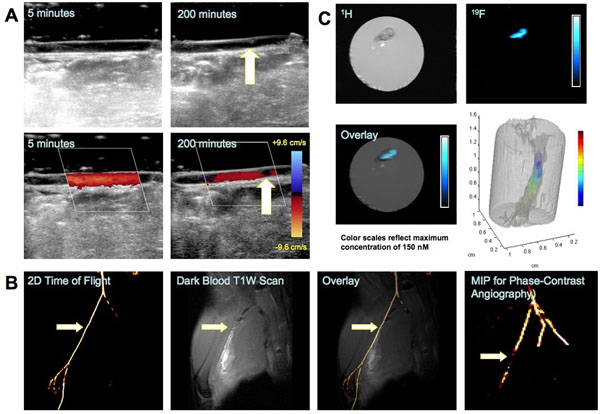


PPACK NPs exceeded PPACK’s activity against thrombin. BVR activity on NPs was insignificantly diminished versus free BVR. Previously, PPACK NPs outperformed both heparin (p=.001) and PPACK (p=.0006) in delaying occlusion of the carotid artery. PPACK or non-functionalized NPs failed to delay occlusion of the carotid artery. BVR NPs significantly delayed occlusion (p=.02) whereas an equivalent dose of free BVR (120 mg/kg) did not (figure 1a). 19F MR captured specific PFC NP retention in occluded arteries (figure 1b), with 19F MRI demonstrating colocalization of particles with the arterial thrombus and quantitative 19F spectroscopy demonstrating specificity of BVR or PPACK NP binding. Ultrasound imaging showed development of thrombi in rabbits (figure 2a). MRA detected the formed thrombus (figure 2b). 19F MRI mapped the thrombus via retention of PPACK NPs.

## Conclusions

Anticoagulant PFC NPs were designed as new antithrombotics with intrinsic magnetic resonance contrast, concentrated therapeutic impact conferred by a thrombin-specific particle surface, and well-defined pharmacokinetics controlled by the particle itself. As potent agents that can enhance the therapeutic performance of different thrombin inhibitors, these NPs are promising agents. Further clinical potential is conferred on the thrombin-inhibiting PFC NP by its ability to guide further intervention by specifically localizing magnetic resonance contrast to sites of thrombosis.

## Funding

This work was supported by National Institutes of Health Grants R01 HL073646 and U54 CA119342 to Samuel Wickline, R01 NS059302 to Gregory Lanza, and R01 HL55520 to Douglas Tollefsen.

